# Comment on Thigpen D. et al. The Role of Ultrasound in Screening Dense Breasts—A Review of the Literature and Practical Solutions for Implementation. *Diagnostics* 2018, *8*, 20

**DOI:** 10.3390/diagnostics8020037

**Published:** 2018-05-24

**Authors:** Wendie A. Berg, JoAnn Pushkin

**Affiliations:** 1 Department of Radiology, University of Pittsburgh School of Medicine, Magee-Womens Hospital of UPMC, Pittsburgh, PA 15213, USA; wendieberg@gmail.com; 2 DenseBreast-info, Inc., Deer Park, NY 11729, USA

We read with interest the article by Thigpen et al. [1]. With 34 states now having some form of density inform legislation [2], issues in implementing supplemental screening are particularly important. There is confusion regarding such legislation, with variable wording from state to state. Six of these laws, including Connecticut as cited in detail by Thigpen et al. (also Maryland, New Jersey, Missouri, Louisiana, and Texas), do not require the mammography facility to inform a woman that she herself has dense breasts. Instead, general notification about breast density is supplied with wording such as “if you have dense breasts”. New Jersey tells all women “you may have dense breasts”. This phrasing leaves a woman uncertain. In 11 states (North Carolina, Nevada, Pennsylvania, Rhode Island, Massachusetts, Delaware, Oklahoma, Vermont, Nebraska, Iowa, and Washington), a woman’s personal breast density (i.e., fatty, scattered, heterogeneously dense, or extremely dense) must be included in the lay letter, but a woman may not know that scattered fibroglandular density is not dense and no translation is required to be provided. In North Carolina, only women with dense breasts (heterogeneously dense or extremely dense) are sent their personal density category, but the letter must be worded with “you may have dense breasts”.

The masking potential of dense tissue to hide detection of breast cancer or an “abnormality” is included in 28 existing laws (not including Iowa’s law which has vague language on this issue or North Dakota’s law which expired 31 July 2017), but only 14 (not including Maine’s “suggested” communication) mention supplemental screening. Even when supplemental screening is mentioned, none of the state laws inform women that adding screening ultrasound or MRI can detect cancer not visible with mammography, as Thigpen et al. suggest.

Successful communication of the increased risk of developing breast cancer and the masking potential associated with dense breast tissue is of great importance and should be considered together with other risk factors. High-risk women of any breast density should be identified and offered screening MRI and possible genetic testing. Tomosynthesis (3D mammography), while an improvement on conventional mammography, has not been shown to improve cancer detection in extremely dense breasts [3]. It is important to provide options to improve cancer detection in the setting of dense breasts. Ultrasound is one such option, though it detects far fewer cancers than MRI [4]. It is equally important for women to understand the chance of a false positive, potentially including biopsy, for suspicious findings on supplemental screening that prove not to be cancer. It is only through such communication that women can decide for themselves if they wish to pursue additional screening beyond mammography/tomosynthesis. We have developed the website, www.DenseBreast-info.org to provide medically sourced information to patients and healthcare providers to assist in such shared decisions.
